# Abstract not submitted for online publication>

**DOI:** 10.1186/1753-6561-6-S3-P16

**Published:** 2012-06-01

## Abstract not submitted for online publication

